# Medical and Physical Intelligence

**Published:** 1806-06

**Authors:** 


### MEDICAL AND PHYSICAL INTELLIGENCE.

The latter end of April, the cow-pox appeared at a farm in Dr. Jenner's neighbourhood in Berkley. The disease was regularly traced from a horse's heel in the farm to the boy who dressed him, and from him to the cows. Dr. Jenner sent notice of this event to Dr. Adams, of the Small Pox Hospital, who, it will be easily supposed, could not lose this opportunity of tracing a morbid poison. Dr. Adams passed the night at Berkley, that the succeeding day might be devoted to their inquiries. In the morning the two physicians, with Mr. Tanner, met at the farm. All the cows were exhibited, the farmer being present, who gave the history of the event with much simplicity, though with tolerable severity when he came to describe the inattention of the boy. The boy afterwards appeared, and urged in his own defence, that he had only milked two of the cows after the injunction he had received. Unfortunately, these were afterwards mixed with the herd, and became affected before they were suspected; in consequence of which, the milkers carried the contagion to all the rest. It was not a little curious to remark those cows who had suffered on a former occasion, and who were easily distinguished by having the disease in a milder form. Dr. Adams brought some of the matter with him to town, and has since used it in the Hospital, to see whether it were possible to distinguish between the effects of matter immediately from the cow, and after it has been for four or five years inserted only in the human subject; but no difference could be perceived.——The visit was made still more interesting by the dog-distemper at that time prevailing in Lord Berkley's farm. The number of animals furnished specimens of every stage of the disease, and in all its forms.

Dr. Thornton has laid before the public some cases, which show the efficacy of vital air, or, as it is usually called, oxygen gas, in the cure of fits. These cases, deemed beyond the reach of human art, have been completely and radically cured by the continued use of the pneumatic medicine. According to the Doctor's theory, vital air gives energy to the muscles, and thence to the nerves, taking off inordinate action from an undue balance of principles. Hence he infers, that persons breathing much bad air become convulsed

Mr. Collard, of Birmingham, has found that copper may be precipitated from its solution in the sulphuric acid by means of tin. The success of the experiment depends upon the heat of the solution, which must be at or near the boiling point, when the tin is put into it. This discovery, it is expected, will lead to some very important results.

Relative to the human phenomenon, Mr. Daniel Lambert, of Leicester, now exhibiting himself in Piccadilly, we have been favoured favoured with the following correct particulars.—This extraordinary man is about 36 years of age; five feet eleven inches high; and his weight is upwards of fifty ftone, fourteen pounds to the ftone. He enjoys perfect health and vigour; his breathing is free and eafy; his fleep undifturbed, to which he has no extraordinary propenfity; and he eats common food, and drinks water only. His extraordinary bulk arifes from an immenfe accumulation of fat within the abdomen, and in the adipofe membrane under the fkin. The tumefaction of the thighs, legs, and feet, is enormous; the arms and hands do not much exceed the ufual proportion in fat perfons. All the functions of the body are in good order. He never felt pain or uneafinefs from the ftretching of the fkin. In the progrefs of its diftention, however, he has four or five times had an eryfipelatous inflammation of the legs, which in a week or two was removed by proper treatment, but has been fucceeded by a fcalinefs and thickening of the fkin. His bulk has increafed gradually from twenty years of age. His father and uncle were both large men; but the weight of either did not exceed thirty ftone.

The Royal College of Surgeons of London, has propofed the following fubjects for the Jackfonian prize for the year 1806. (1) The difeafes of joints, particularly of the hip and the knee, and the beft mode of treatment. (2) Herniæ and the beft mode of treatment.

Mr. Royston is engaged in an extenfive work on the Medical Literature of England; with the firft part of which he expects to go to prefs in a few weeks. The object of this work is to give a defcription and analyfis of books publifhed by Englifhmen, on the fcience of medicine; beginning with the earlieft printed works, and ending with the year 1800. It is intended to be given in the manner of a Bibliotheca, defcribing the form and peculiarities of every work, under the fize, *princeps* and *optime* editions, &c. &c. To which an analyfis of the contents of each volume will be added; conftituting a concordance of facts and opinions, arranged in a manner that will afford a ready reference for the ftudent, the practitioner, and the man of fcience.

Mr. Gunning, Surgeon Extraordinary to H. R. H. the Duke of Sussex, and Surgeon to St. George's Hospital, will commence in October next, at his house in Clifford Street, a course of lectures on the Principles and Practice of Surgery. Any gentleman who wishes to have a private course of lectures, on the Operations of Surgery, may know Mr. Gunning's terms by applying to him, in Clifford Street; or at St. George's Hospital.

Dr. Beddoes has in the press, a Report from an Institution at Bristol for investigating the origin and cutting short the progress of consumption, scrophula, and other prevalent disorders in families and individuals. These cases have been kept for several years by various medical gentlemen, who will be named, as well as by the Editor, who will accompany them occasionally by some practical observations.

On

On Monday the 2d of June, a Course of Lectures on Physic and Chemistry, will re-commence at the Laboratory, Whitcomb Street, Leicester Square, at the usual morning hours; viz. Therapeutics at a quarter before eight, the Practice at half after, and the Chemistry at a quarter after nine; by GEORGE PEARSON, M. D. F. R. S. Senior Physician to St. George's Hospital, &c. The Practice of Vaccination will be taught at the Institution, No. 44, Broad Street, Golden Square, according to the usual plan. Further particulars may be had at St. George's Hospital, or at 52, Leicester Square.

Dr. WILLAN's work on the Cow-pox, which we announced in our last number, will comprize the following sections, the titles of which express the subjects to be considered. 1. On the combined inoculation of the variolous and vaccine fluids. 2. On the characteristics and effects of perfect vaccination. 3. On imperfect vaccination. 4. On the cases of small pox subsequent to vaccination. 5. On the cutaneous and glandular diseases *imputed* to vaccine inoculation. 6. On the chicken-pox and swine-pox. 7. On the inoculation of the chicken pox. 8. On the extermination of the small-pox.—The Appendix consists of Letters from Dr. Jenner, and other physicians and surgeons in the principal towns of Great Britain and Ireland.

The Medical Students of St. Thomas's and Guy's Hospitals have presented to Mr. SANDERS, the Demonstrator of Practical Anatomy, a piece of plate of considerable value, as a testimony of their estimation of his attentions to their improvement.

